# New Perspectives in the Chemistry of Marine Pyridoacridine Alkaloids †

**DOI:** 10.3390/md14020026

**Published:** 2016-01-26

**Authors:** Alois Plodek, Franz Bracher

**Affiliations:** Department of Pharmacy—Center for Drug Research, Ludwig-Maximilians University, Butenandtstr. 5-13, 81377 Munich, Germany; loisl.plodek@cup.uni-muenchen.de

**Keywords:** marine alkaloids, pyridoacridines, total synthesis

## Abstract

Secondary metabolites from marine organisms are a rich source of novel leads for drug development. Among these natural products, polycyclic aromatic alkaloids of the pyridoacridine type have attracted the highest attention as lead compounds for the development of novel anti-cancer and anti-infective drugs. Numerous sophisticated total syntheses of pyridoacridine alkaloids have been worked out, and many of them have also been extended to the synthesis of libraries of analogues of the alkaloids. This review summarizes the progress in the chemistry of pyridoacridine alkaloids that was made in the last one-and-a-half decades.

## 1. Introduction

**Figure 1:**
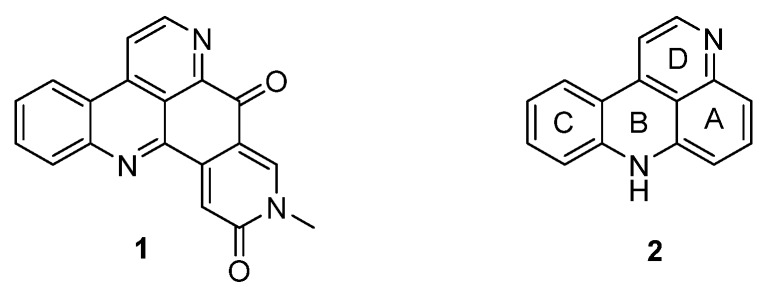
Structure of amphimedine (**1**) and the 11*H*-pyrido[4,3,2-*mn*]acridine scaffold (**2**).

Pyridoacridine alkaloids are a fascinating and emerging class of polycylic alkaloids derived from sessile marine invertebrates such as ascidians, sponges, mollusks, and sea anemones [1]. The era of this alkaloid family started in 1983 with the isolation and identification of amphimedine (**1**) from a pacific sponge (*Amphimedon* sp.) by Shoolery [2]. Since then, more than 100 of these polycyclic heteroaromatic natural compounds have been isolated and, according to the biosynthetic considerations of Skyler and Heathcock, a large number of “undiscovered“ alkaloids is assumed [3]. Pyridoacridines are highly colored marine metabolites and are characterized by the 11*H*-pyrido[4,3,2-*mn*]acridine skeleton (**2**) [4] (Figure 1). Besides a few hepta- and octacyclic members, tetra-, penta-, and hexacyclic compounds form the largest subgroup of pyridoacridine alkaloids [3].

The pyridoacridines and numerous analogues have been reported to possess manifold biological activities. The outstanding biological effect in this context is beyond doubt their high cytotoxicity. Various mechanisms of action have been described for these anticancer activities. The predominant effects are inhibition of topoisomerase II and the formation of reactive oxygen species (ROS). Furthermore, several pyridoacridines and analogues are associated with antimicrobial, insecticidal and antiparasitic acitivities against *Plasmodium*, *Leishmania*, and *Trypanosoma* species [1]. Consequently, this alkaloid family is of great interest as a source of new lead structures in medicinal chemistry.

The pharmacology of pyridoacridine alkaloids has been reviewed comprehensively very recently [5].

Since only minute amounts of these alkaloids can be obtained from natural sources, total synthesis is an indispensable means for development of drugs from this chemotype. On the other hand, effective strategies for the total syntheses of the alkaloids also open the opportunity for preparing synthetic analogues of the natural products for getting deeper insight into structure-activity relationships and improving pharmacokinetic and pharmacodynamic properties.

Several reviews on the chemistry of pyridoacridine alkaloids have been published over the years [4], and the most recent one was authored by Delfourne in 2002 [6]. The present review represents an update of Delfourne’s compilation, including some older work that is missing in [6].

## 2. Ascididemin-Type Pyridoacridines

**Figure 2:**
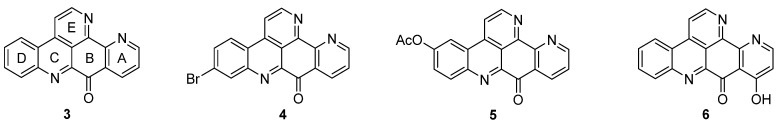
Structures of the ascididemin-type pyridoacridine alkaloids: ascididemin (**3**), bromoleptoclinidinone (**4**), neocalliactine acetate (**5**), 10-hydroxyascididemin (**6**).

Ascididemin (**3**) and closely related pyridoacridine alkaloids (bromoleptoclinidinone (**4**), neocalliactine acetate (**5**), and 10-hydroxyascididemin (**6**)) share the same pentacyclic ring system, in which a pyridine ring is annulated to ring A of the 11*H*-pyrido[4,3,2-*mn*]acridine scaffold (**2**) (Figure 2).

**Scheme 1:**
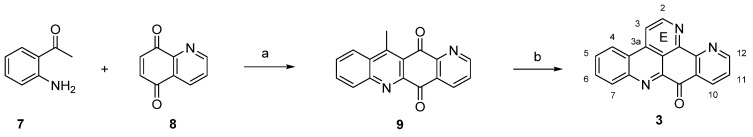
First total synthesis of ascididemin (**3**): (**a**) CeCl_3_·7H_2_O, EtOH, air; then conc. H_2_SO_4_/AcOH (73% over two steps); (**b**) *N*,*N*-dimethylformamide diethyl acetal, DMF; then NH_4_Cl, AcOH (59% over two steps).

The first and still-leading synthesis of the alkaloid acsididemin (**3**) was published by Bracher in 1989 [7]. Albeit being presented in numerous reviews before, this synthesis is shown here once again, since its crucial steps have found application in several of the more recent approaches to diverse pyridoacridine alkaloids that will be presented below. The synthesis starts with an oxidative amination of 2-aminoacetophenone (**7**) and quinoline-5,8-dione (**8**), followed by an acid-catalyzed cyclization step to give tetracyclic quinone **9**. The final annulation of ring E was performed in a one-pot reaction by condensation of the acidic methyl group with *N*,*N*-dimethylformamide diethyl acetal, followed by heating with ammonium chloride/glacial acetic acid to give **3** [7] (Scheme 1). This high-yielding four-step approach (43% overall yield) has later been applied to the total synthesis of many related pyridoacridines and analogues thereof by simply using pertinent ring-substituted 2-aminoacetophenones as starting materials [8,9,10,11].

**Scheme 2:**
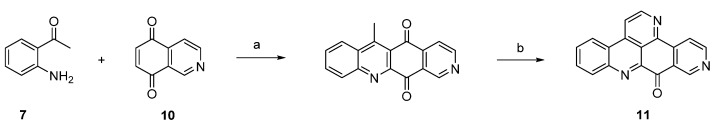
Synthesis of ascididemin isomer **11**. Reagents and conditions: (**a**) CeCl_3_·7H_2_O, EtOH, air; then conc. H_2_SO_4_/AcOH (27% over two steps); (**b**) *N,N*-dimethylformamide diethyl acetal, DMF; then NH_4_Cl, AcOH (23% over two steps).

The isomer **11** of ascididemin, in which the nitrogen in ring A is shifted from position 13 to 11, was prepared by the Delfourne group [12] in full analogy to Bracher’s method, with a surprisingly regioselective oxidative amination (51% yield) of isoquinoline-5,8-dione (**10**) and 2-aminoacetophenone (**7**) as the key step (Scheme 2).

Based on Bracher’s synthetic strategy [7], several modified approaches towards ascididemin-type pyridoacridines have been developed. Most of these syntheses aim at the replacement of the 2-aminoacetophenone building block by more complex 2-substituted anilines, which bear a residue that introduces not only C-3 and C-3a of the final pentacycle (as the acetyl residue does), but contain, in addition, equivalents of the later C-2 an the ring nitrogen of ring E (for numbering of the ring system, see Scheme 1). In earlier approaches, *N*-trifluoroacetamidokynuramine (bearing a protected 3-aminopropanoyl side-chain) [13] and 2-aminocinnamaldehyde-*N*,*N*-dimethylhydrazone [14] were used for this purpose.

In a very recent syntheses of various ascididemin-type alkaloids, consisting of the natural products ascididemin (**3**), bromoleptoclinidinone (**4**), neocalliactine acetate (**5**), 10-hydroxyascididemin (**6**), as well as two synthetic analogues 5-methoxyascididemin (**17**) and deazaascididemin (**18**), Yin *et al.* introduced a novel C_3_N unit, the Boc-protected propargylamine group, for this purpose [15].

**Scheme 3:**
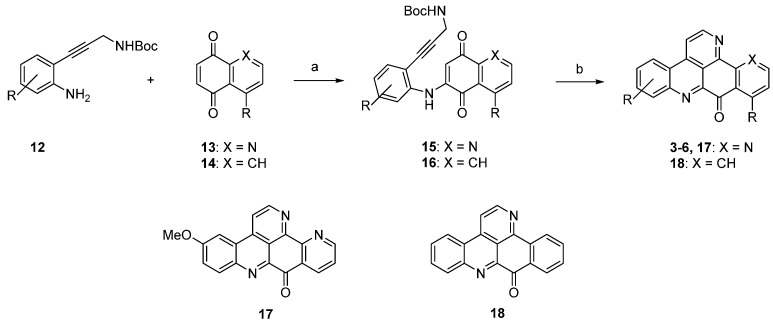
Synthesis of pyridoacridine natural products **3**–**6** and synthetic ascididemin analogues 5-methoxyascididemin (**17**) and deazaascididemin (**18**): (**a**) CeCl_3_·7H_2_O, MeOH, O_2_ (50%–80%); (**b**) Fe_2_(SO_4_)_3_, conc. H_2_SO_4_/AcOH, O_2_ (44%–86%).

The required alkyne building blocks **12** were synthesized via Sonogashira reaction of the alkyne *N*-Boc-propargylamine and a variety of 2-iodoanilines. In analogy to the original synthesis [7], this approach started with an oxidative amination step of alkynylanilines **12** with quinones **13** and **14**. The so-obtained arylaminoquinones **15** and **16** were transferred to alkaloids **3**–**6**, and analogues **17** and **18** via a Brønsted acid-promoted tandem annulation in very good yields (Scheme 3) [15].

As a number of investigations on the biological activities of ascididemin-type pyridoacridines indicated that cytotoxic activity and selectivity (e.g., against human and protozoal cells) is strongly dependent on the shape of ring A (especially the presence and position of heteroatoms in this ring) [5,16,17,18,19], numerous investigations aimed at working out novel and flexible approaches to ring A analogues of ascididemin (**3**) were undertaken in the past decade.

**Scheme 4:**
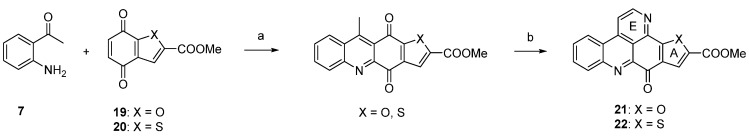
Synthesis of ascididemin-type pyridoacridines **21** and **22** by Copp: (**a**) CeCl_3_·7H_2_O, MeOH, air (22%–92%); then conc. H_2_SO_4_/AcOH (81%–94%); (**b**) NH_4_Cl, (CH_2_O)*_n_*, AcOH (76%–83%).

The approach described by Copp in 2010 [18] in general follows Bracher’s strategy [7], but the authors utilized thiophene and furan quinones **19** and **20** instead of quinoline-5,8-dione (**8**) in order to introduce heteroatom-containing five-membered ring A substitutes. The final annulation of ring E (under concomitant rearomatization) was performed with paraformaldehyde and ammonium chloride to give ascididemin-type pyridoacridines **21** and **22** (Scheme 4) [18].

**Scheme 5:**
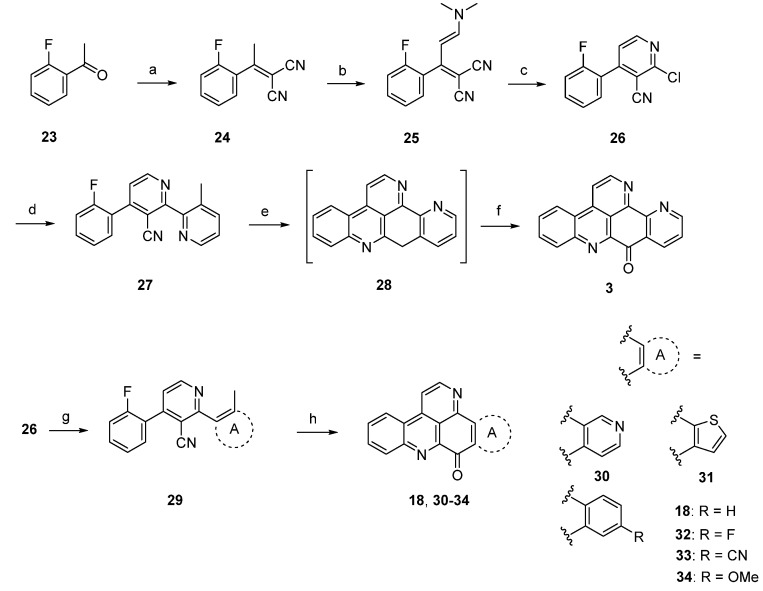
Synthesis of ascididemin (**3**) through anionic ring closure: (**a**) Malononitrile, NH_4_OAc, toluene/AcOH; (**b**) dimethylformamide dimethyl acetal, CH_2_Cl_2_; (**c**) HCl gas, AcOH (81% over three steps); (**d**) 3-methylpyridin-2-ylzinc bromide, PEPPSI-iPr, THF (80%); (**e**) NaH, DMF; (**f**) O_2_ (69% over two steps) [20]; Synthesis of the ring A analogues **18**, **30**–**34**; (**g**) Negishi or Suzuki cross-coupling reactions (44%–88%); (**h**) NaH, DMPU (19%–29%).

A completely different approach towards ascididemin (**3**) and its analogues using an anionic ring closure as the key step was published by Kristensen in 2012 [20]. Synthesis of ascididemin (**3**) started with a Knoevenagel condensation of 2’-fluoroacetophenone (**23**) and malononitrile. The CH-acidic methyl group in the resulting product **24** was condensed with *N*,*N*-dimethylformamide dimethyl acetal to give enamine **25**. Upon exposure of **25** to HCl in glacial acetic acid cyclization to the 2-chloropyridine, **26** was accomplished. Negishi cross-coupling with 3-methylpyridin-2-yl zinc bromide under PEPPSI-iPr catalysis gave the bipyridine **27**, the central precursor for the anionic cascade. Treating **27** with NaH in DMF under microwave irradiation furnished 9-deoxyascididemin (**28**) via an anionic ring closure cascade. As **28** proved to be very difficult to handle, the crude intermediate was directly oxidized by bubbling oxygen through the solution. The desired alkaloid **3** was finally isolated in 45% overall yield (Scheme 5). An isomer of the alkaloid, prepared by using 4-methylpyridin-3-yl zinc bromide was obtained in the same manner in comparable overall yield.

Based on this methodology, several ring A analogues of ascididemin **18**, **30**–**34** were prepared (Scheme 5) [21]. Different ring A equivalents were introduced via Neghishi or Suzuki cross-coupling reactions to give biaryls **29**. The following anionic ring closure was achieved here by using NaH in *N*,*N*’-dimethylpropylene urea (DMPU). However, the yields of the final cyclization cascade were rather low (12%–29%) here, and several target compounds bearing heteroarenes as ring E equivalents (among them the annulated thiazole kuanoniamine A) could not be obtained at all.

**Scheme 6:**
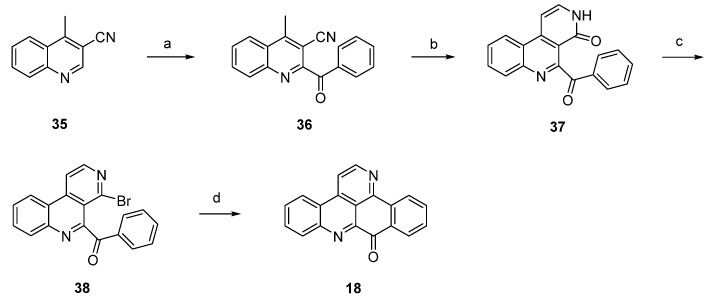
Synthetic approach towards deazaascididemin (**18**): (**a**) Benzaldehyde, AcOH, Et_3_N, H_2_O, FeSO_4_, *tert*-BuOOH (51%); (**b**) dimethylformamide diethyl acetal, DMF; then H_2_SO_4_, AcOH (39%); (**c**) POBr_3_, anisole (27%); (**d**) Bu_3_SnH, AIBN, toluene (5%).

In 2012, Raeder and Bracher published a new synthetic approach to the pyridoacridine ring system involving two radical reactions as the key steps [22]. Readily available quinoline **35** was subjected to Minisci benzoylation with a benzoyl radical generated from benzaldehyde to furnish ketone **36**. The initially low yield of this step was increased by replacing sulfuric acid by trifluoroacetic acid and by adding additional amounts of radical starters (iron(II) sulfate, *tert*-butyl hydroperoxide) after intervals of 45 min. The annulation of the bromopyridine ring, which later forms ring E of the target compound, was accomplished in a two-step protocol. First, **36** was condensed with dimethylformamide diethyl acetal, followed by heating with concentrated sulfuric acid/glacial acetic acid to give pyridone **37**. In the second step, **37** was converted to bromo compound **38** by heating with phosphoryl bromide. The final intramolecular cyclization was performed through conversion of the bromopyridine moiety to an aryl radical with tributyltin hydride and azobis(isobutyronitrile) (AIBN) to give deazaascididemin (**18**) (Scheme 6). Unfortunately, this approach gives only a very low overall yield, and the scope of this route has not been explored.

**Scheme 7:**
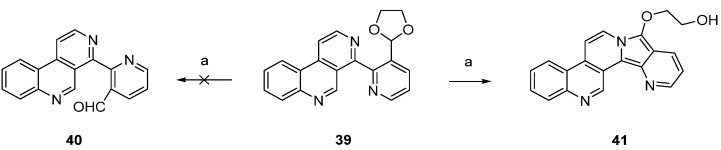
Unsuccessful approach to the ascididemin precursor **40** giving benzo[*f*]pyrido[2′,3′:3,4]pyrrolo[2,1-*a*][2,7]naphthyridine (**41**): (**a**) H_2_SO_4_, H_2_O (43%).

In a related approach, Bracher [23] intended to utilize the Minisci reaction as a final cyclization step for the synthesis of ascididemin (**3**). However, attempted acidic hydrolysis of dioxolane **39** (to give aldehyde **40** as an acyl radical precursor) resulted in unexpected cyclization to the benzo[*f*]pyrido[2′,3′:3,4]pyrrolo[2,1-*a*][2,7]naphthyridine **41** (Scheme 7).

**Scheme 8:**
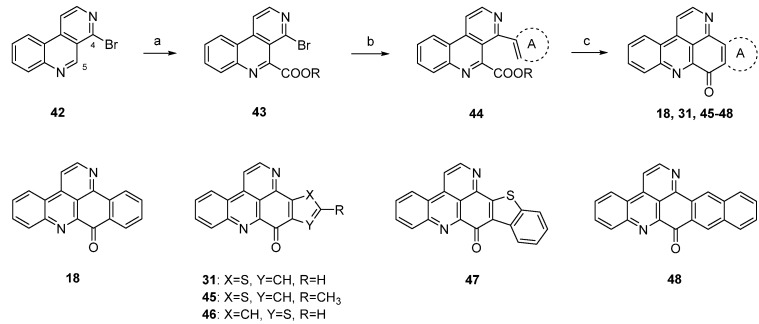
Synthesis of ring A analogues of ascididemin **18**, **31**, **45**–**48** through an intramolecular trifluoromethanesulfonic acid-aided Friedel-Crafts-type cyclization step: (**a**) Methyl pyruvate, H_2_O_2_, FeSO_4_, H_2_SO_4_, AcOH; then MnO_2_, CH_2_Cl_2_ (93% over two steps); (**b**) (hetero)areneboronic acid, Pd(Ph_3_P)_4_ (cat.), K_2_CO_3_, THF, H_2_O (41%–75%); (**c**) CF_3_-SO_3_H, microwave irradiation (63%–92%).

A different synthetic approach to ring A analogues of ascididemin from the Bracher group [24] started with a high-yielding, regioselective Minsici-type homolytic methoxycarbonylation at C-5 of readily available 4-brombenzo[*c*][2,7]naphthyridine (**42**), followed by an introduction of ring A equivalents through Suzuki cross-coupling with appropriate (hetero)areneboronic acids. The final intramolecular ring closure was achieved by treating the 4,5-disubstituted benzo[*c*][2,7]naphthyridines **44** with trifluoromethanesulfonic acid under microwave irradiation. This final superacid-aided Friedel-Crafts-type acylation step furnished pyridoacridines **18**, **31**, **45**–**48** in 63%–92% yields (Scheme 8). Significantly lower yields were obtained with the corresponding ethyl esters. However, these cyclization reactions were successful only with electron-rich aryl residues (phenyl, naphthyl, thiophenes) in position 4. Electron-withdrawing substituents (acetyl, chloro, bromo) on a benzene or thiophene ring led to the complete failure of this cyclization step [24].

**Scheme 9:**
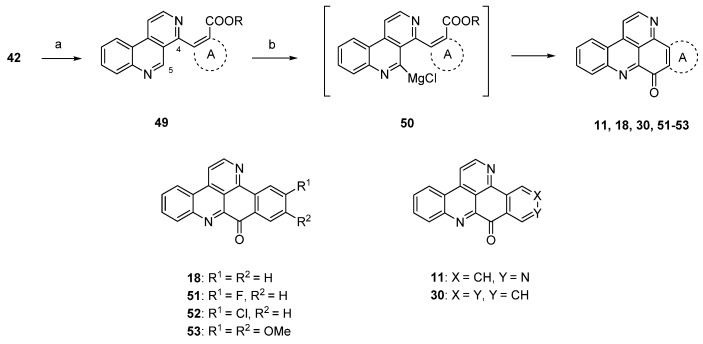
TMPMgCl·LiCl-mediated synthesis of ring A analogues **18**, **31**, **45**–**48** and isomers **11** and **30** of ascididemin (**3**): (**a**) For Suzuki cross-coupling reactions: areneboronic acid, Pd_2_(dba)_3_, P(*t*Bu)_3_, KF, THF (73%–80%); for Negishi cross-coupling reactions: pyridylzinc compounds, Pd(dba)_2_, P(2-furyl)_3_, THF (71%–76%); (**b**) TMPMgCl·LiCl, THF (27%–39%).

Due to the fact that the above-mentioned protocol [24] only allows for the introduction of electron-rich carbocyclic and heterocyclic ring A substitutes, Plodek *et al.* developed a new approach to the pyridoacridine ring system in which electron-deficient (hetero)arenes also serve as sources for ring A [25]. The introduction of the ring A scaffolds was achieved through Suzuki cross-coupling of 4-brombenzo[*c*][2,7]naphthyridine (**42**) with (hetero)areneboronic acids bearing an ester moiety in the *ortho* position or through Negishi cross-coupling with pyridylzinc compounds which were obtained by regioselective ring metalation of ethyl nicotinate or ethyl isonicotinate. The resulting 5-substituted benzo[*c*][2,7]naphthyridines **49** were metalated regioselectively at the *peri* position (C-5) with Knochel’s TMPMgCl·LiCl (2.2 equivalents), and intramolecular nucleophilic attack of the resulting arylmagnesium species **50** at the ester group furnished ring A analogues **18**, **31**, **45**–**48** and isomers **11** and **30** of ascididemin in poor-to-modest yields (Scheme 9) [25]. This key step was inspired by the synthesis of demethyldeoxyamphimedine published by the Bracher group in 2014 [26] (see below).

The protocol shown in Scheme 9 is the most flexible one for the preparation of ring A analogues of ascididemin (**3**), which are of special interest for the development of anticancer compounds.

## 3. Amphimedine-Type Pyridoacridines

**Figure 3:**
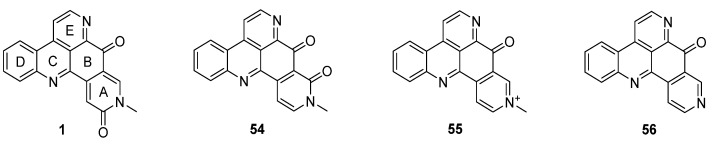
Structures of the amphimedine-type pyridoacridine alkaloids: amphimedine (**1**), neoamphimedine (**54**), deoxyamphimedine (**55**) and demethyldeoxyamphimedine (**56**).

Amphimedine-type pyridoacridines consist of the pentacyclic alkaloids amphimedine (**1**), neoamphimedine (**54**), deoxyamphimedine (**55**), and demethyldeoxyamphimedine (**56**) (Figure 3) [26]. Compared to the ascididemin subclass, rings A and B are connected in a different manner here. While most of the published syntheses of ascididemin-type pyridoacridines are more or less based on Bracher’s synthetic methodology [7], many different and versatile strategies towards the amphimedine scaffold have been developed [6]. During the last 15 years, three approaches towards neoamphimedine (**54**) and two syntheses of demethyldeoxyamphimedine (**56**) have been reported.

**Scheme 10:**
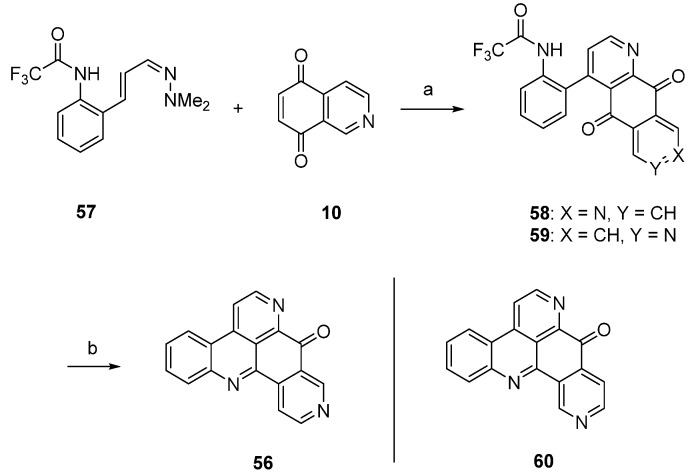
Synthesis of demethyldeoxyamphimedine (**56**): (**a**) Toluene, N_2_ atmosphere (0.8%); (**b**) NaOH, CHCl_3_ (96%).

Delfourne’s first total synthesis of demethyldeoxyamphimedine (**56**), which is illustrated in Scheme 10, was published in 2002 [12], years before this compound was identified as a natural product [27]. This approach started with a hetero-Diels-Alder cycloaddition of isoquinoline-5,8-dione (**10**) and 1-azadieene **57**, both of them not commercially available. Besides the desired diazaanthranquinone **58**, which was isolated in only 0.8% yield, the poorly separable regioisomer **59** was formed in 1.7% yield. The final ring closure step was accomplished under alkaline conditions to give demethyldeoxyamphimedine (**56**) in almost quantitative yield. Analogous cyclization of the isomeric intermediate **59** gave the isomer **60** of the alkaloid in 91% yield.

**Scheme 11:**
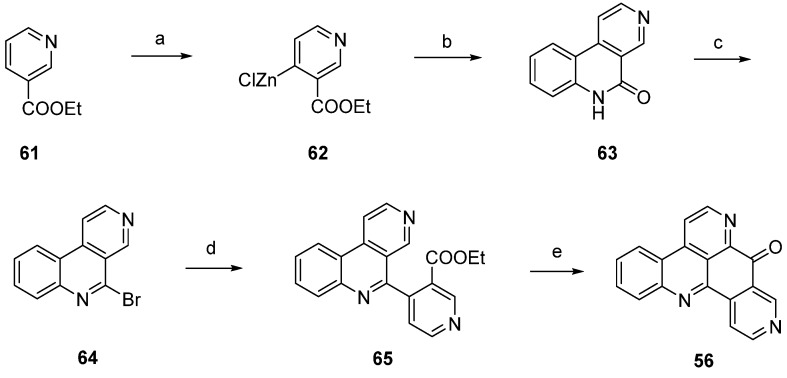
Synthesis of demethyldeoxyamphimedine (**56**) by Melzer *et al*: (**a**) TMPMgCl·BF_3_·LiCl, THF; then ZnCl_2_; (**b**) 2-iodoaniline, Pd(dba)_2_, P(2-furyl)_3_, THF (50% over two steps); (**c**) POBr_3_ (59%); (**d**) pyridylzinc compound 62, Pd(dba)_2_, P(2-furyl)_3_, THF (78%); (**e**) TMPMgCl·LiCl, THF (28%).

A highly effective total synthesis of demethyldeoxyamphimedine (**56**) was reported by Melzer *et al.* in 2014 [26]. Regioselective direct metalation of ethyl nicotinate (**61**) at C-4 using TMPMgCl·BF_3_·LiCl and subsequent transmetalation with ZnCl_2_ gave pyridylzinc compound **62**, which was directly subjected to a Negishi cross-coupling reaction with 2-iodoaniline. The resulting biaryl (not shown in Scheme 11) underwent spontaneous lactamization to give benzo[*c*][2,7]naphthyridin-5(6*H*)-one (**63**). This lactam was converted to 5-bromobenzo[*c*][2,7]naphthyridine (**64**) with phosphoryl bromide. In another Negishi cross-coupling reaction, tricyclus **64** was coupled with organozinc intermediate **62** to give biaryl **65**. The final ring closure step was achieved via direct regioselective metalation at C-4 of **65** utilizing 2.2 equivalents of Knochel’s TMPMgCl·LiCl, followed by intramolecular trapping of the ester group to give alkaloid **56** in 6.4% total yield. Thus, the authors prepared demethyldeoxyamphimedine (**56**) by using only two commercial building blocks, ethyl nicotinate and 2-iodoaniline (Scheme 11) [26].

**Scheme 12:**
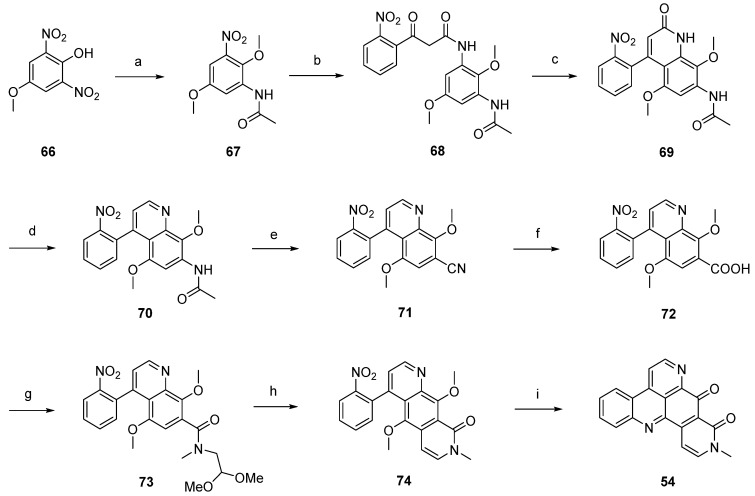
Ireland’s synthesis of neoamphimedine (**54**): (**a**) CH_2_N_2_; then 10% Pd/C in cyclohexene/EtOH; then AcOH/Ac_2_O (77% over two steps); (**b**) 10% Pd/C in cyclohexene/EtOH; then ethyl (2-nitrobenzoyl)acetate, *m*-xylenes (96%); (**c**) PPA (52%); (**d**) Tf_2_O, CH_2_Cl_2_, Et_3_N; then formic acid, Et_3_N, DMF, Pd(OAc)_2_, dppf (61% over two steps); (**e**) AcOH, H_2_O, H_2_SO_4_; then NaNO_2_; then CuCN (50%); (**f**) H_2_SO_4_ (80%); (**g**) *N*-methylamino acetaldehyde dimethyl acetal, EDC, CH_2_Cl_2_ (87%); (**h**) H_2_SO_4_ (43%); (**i**) 10% Pd/C, cyclohexene/EtOH; then CAN (30%).

The first total synthesis of neoamphimedine (**54**) was reported by Ireland in 2007 [28]. This approach started with *O*-methylation of dinitrophenol **66** with diazomethane, followed by selective reduction of one of the two nitro groups with 10% palladium on carbon and subsequent acetylation of the so-obtained amine (not shown in Scheme 12) with acetic anhydride to afford acetanilide **67**. Catalytic reduction and following conversion with ethyl (2-nitrobenzoyl)acetate furnished β-keto amide **68** in very good yields. Treatment of **68** with polyphosphoric acid gave quinolone **69** in a Knorr cyclization reaction. Quinoline **70** was obtained in a two-step protocol through treating **69** with trifluoromethanesulfonic anhydride and subsequent hydrogenolysis of the resulting triflate ester. The following transformation of the 7-acetylamino group of **70** into a nitrile was accomplished under Sandmeyer conditions after acidic hydrolysis. Next, the nitrile of **71** was hydrolyzed by heating with concentrated sulfuric acid to give the carboxylic acid **72**, and further amidated with *N*-methylamino acetaldehyde dimethylacetal to give carboxamide **73**. Pomeranz-Fritsch–type cyclization with sulfuric acid gave oxo-diazaanthracene **74**. The desired alkaloid **54** was obtained by catalytic reduction of the nitro group of **74**, subsequent CAN oxidation to a tricyclic quinone, followed by intramolecular condensation with the amino group in a total yield of 2% over 12 steps (Scheme 12) [28].

**Scheme 13:**
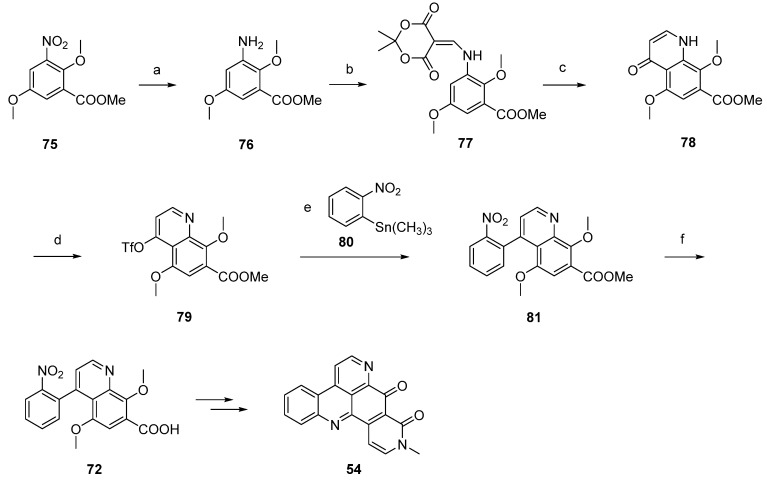
Improved synthesis of neoamphimedine (**54**): (**a**) Pd/C, H_2_, MeOH; (**b**) Meldrum’s acid, trimethyl orthoformate (90% over two steps); (**c**) Ph_2_O, reflux (87%); (**d**) Tf_2_O, DMAP, 2,6-lutidine, CH_2_Cl_2_ (92%); (**e**) trimethyl-(2-nitrophenyl)stannane (**80**), CuI, Pd(OAc)_2_, dppe, DMF (83%); (**f**) LiOH; following steps, see Scheme 12.

Based on this methodology, LaBarbera published an improved total synthesis of neoamphimedine (**54**) [29]. The central quinolone intermediate **77** was synthesized in three steps, starting from nitrobenzoate **75**. Catalytic hydrogenation of **75** gave the aniline **76**, which was converted to **77** by treatment with Meldrum’s acid and trimethyl orthoformate. The following thermal ring closure gave 4-quinolone **78** with an overall yield of 78%. Treatment of **78** with trifluoromethanesulfonic anhydride furnished triflate ester **79**, which was smoothly converted to the biaryl **81** in a Stille cross-coupling reaction with readily available trimethyl-(2-nitrophenyl)stannane (**80**). Next, alkaline hydrolysis of the methyl ester group gave carboxyclic acid **72**. The following steps were performed as described above [28] and afforded neoamphimedine (**54**) in 25% overall yield (Scheme 13) [29].

**Scheme 14:**
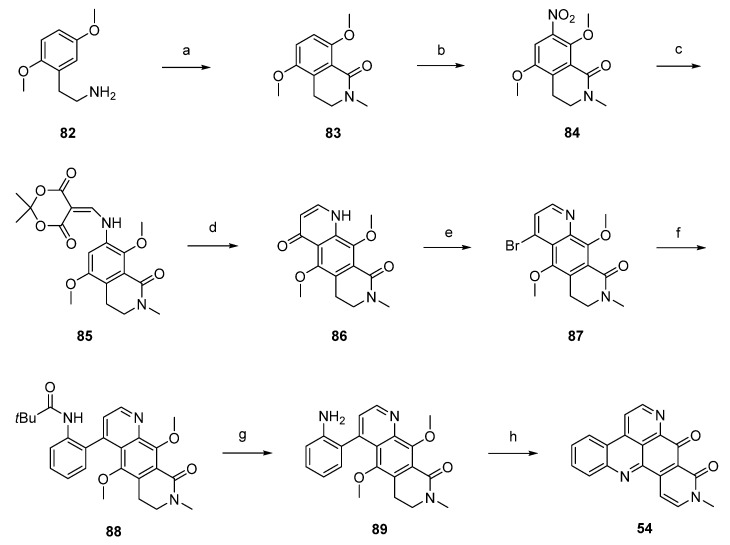
Synthesis of neoamphimedine (**54**): (**a**) ClCO_2_Et, Et_3_N, THF; then MeI, NaH, THF; then DMAP, (CF_3_SO_2_)O, CH_2_Cl_2_ (74% over three steps); (**b**) Cu(NO_2_)_2_∙3H_2_O, Ac_2_O (96%); (**c**) Pd/C, H_2_, MeOH; then Meldrum’s acid, trimethyl orthoformate (81% over two steps); (**d**) Ph_2_O, reflux (83%); (**e**) POBr_3_, THF (70%); (**f**) 2-(pivaloylamino)phenylboronic acid, Pd(PPh_3_)_4_, K_2_CO_3_, H_2_O, EtOH, toluene (90%); (**g**) H_2_SO_4_/H_2_O (64%); (**h**) BBr_3_, CH_2_Cl_2_; then CAN/H_2_O, MeCN (31%).

Another synthetic approach to neoamphimedine (**54**) was reported by Nakahara *et al.* in 2012 [30]. Commercially available 2,5-dimethoxyphenethylamine (**82**) was *N*-acylated with ethyl chloroformate and triethylamine. *N*-Methylation of the so-obtained *N*-carbethoxy derivative (not shown in Scheme 14) was performed with methyl iodide and sodium hydride. Subsequent Bischler-Napieralski–type cyclization with triflic anhydride and *N*,*N*-dimethylaminopyridine (DMAP) furnished quinolone **83** in 74% overall yield. Following regioselective nitration of **83** with cupric nitrate trihydrate in acetic anhydride afforded **84** in excellent yield. Catalytic hydrogenation of the nitro group utilizing 10% palladium on carbon and subsequent reaction with Meldrum’s acid and trimethyl orthoformate yielded enamine **85**, which was cyclized to acridinedione **86** by heating in diphenyl ether. Subsequent bromination with phosphoryl bromide afforded **87**, and Suzuki cross-coupling with 2-(pivaloylamino)phenylboronic acid gave intermediate **88** in high yield. Acid-mediated removal of the pivaloyl protective group gave amine **89**. Conversion to the alkaloid **54** was performed in two steps: *O*-demethylation with BBr_3_ gave a hydroquinone intermediate which underwent cyclization and dehydrogenation in the dihydropyridone ring upon oxidation with CAN (31% yield over both steps). Using nitric acid as the oxidant led to a significant loss in yield (6%). The total yield of neoamphimedine (**54**) over 12 steps was 6% (Scheme 14) [30].

## 4. Eilatin-Type Pyridoacridines

**Figure 4:**
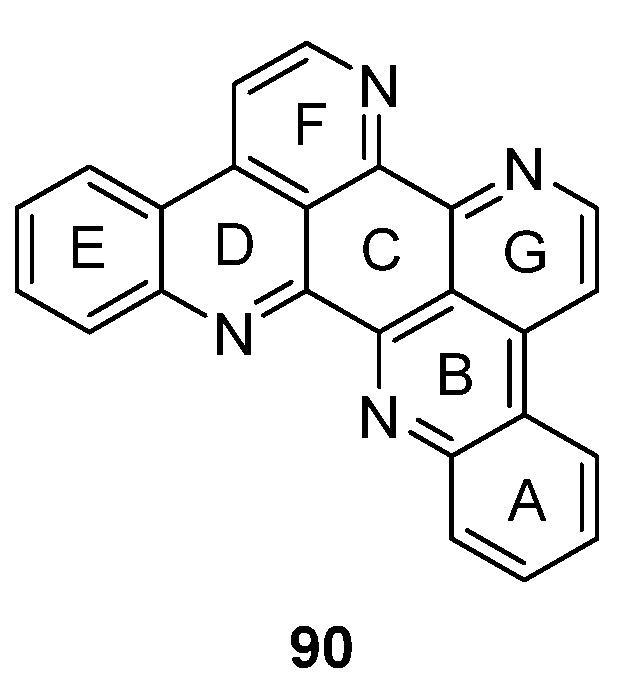
Structure of eilatin (**90**).

The symmetrical alkaloid eilatin (**90**, Figure 4) is the only known heptacyclic member of the pyridoacridine alkaloid class and was isolated in 1988 by Rudi *et al.* from the tunicate *Eudistoma* sp. [31]. Since synthetic approaches towards eilatin (**90**) have not been reviewed before, this chapter will close this gap and gives a short overview on all published syntheses since its isolation.

**Scheme 15:**
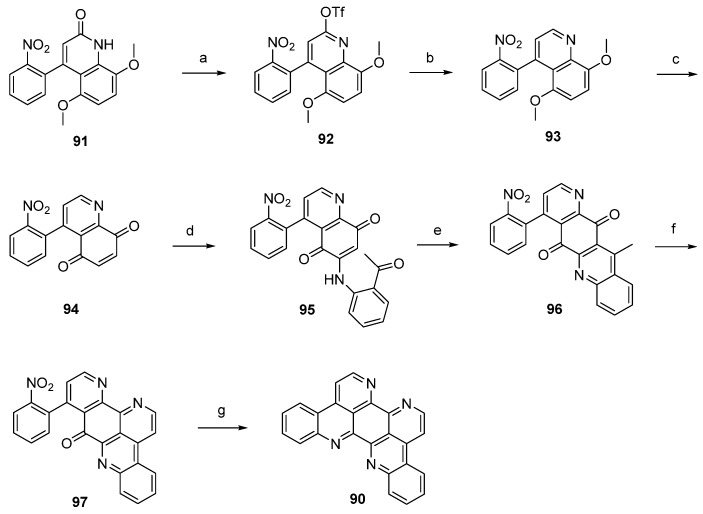
Synthesis of eilatin (**90**): (**a**) Tf_2_O, CH_2_Cl_2_, Et_3_N (93%); (**b**) formic acid, Et_3_N, Pd(OAc)_2_, dppf, DMF (87%); (**c**) CAN, acetonitrile/H_2_O (60%); (**d**) CeCl_3_·7H_2_O, 2-aminoacetophenone, EtOH (54%); (**e**) conc. H_2_SO_4_/AcOH (83%); (**f**) *N*,*N*-dimethylformamide diethyl acetal, DMF; then NH_4_Cl, AcOH (75% over two steps); (**g**) 10% Pd/C, EtOH (85%).

The first total synthesis of eilatin (**90**), which is illustrated in Scheme 15, was reported by the Kubo group in 1993 [32]. The alkaloid was obtained in seven steps (overall yield 13%) from intermediate **91**, which had been developed before as an intermediate for a total synthesis of amphimedine (**1**) [33]. Arylquinolone **91** was converted to the aryl triflate **92** with trifluoromethanesulfonic anhydride, and subsequent palladium-catalyzed reductive removal of the sulfonyloxy group with triethylammonium formate furnished 4-arylquinoline **93**. Following oxidative demethylation of **93** using CAN gave *p*-quinone **94**. The next three steps follow Bracher’s ascididemine synthesis (see Scheme 1), thus arylaminoquinoline-5,8-dione **95** was prepared by regioselective oxidative amination of *p*-quinone **94** with 2-aminoacetophenone in the presence of CeCl_3_. Next, an acid-catalyzed cyclization step was performed furnishing tetracyclic quinone **96**, and the annulation of ring G was achieved using condensation with *N*,*N*-dimethylformamide diethyl acetal followed by ammonium chloride treatment to afford pentacyclus **97**. The final ring closure step was accomplished through catalytic hydrogenation of the nitro compound **97** with 10% palladium on carbon and spontaneous cyclocondensation to give eilatin (**90**) (Scheme 15) [32].

**Scheme 16:**
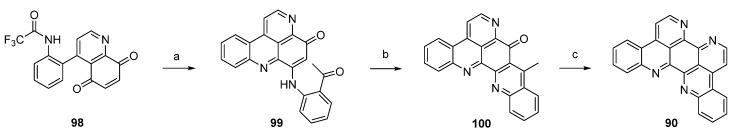
Synthesis of eilatin (**90**): (**a**) CeCl_3_·7H_2_O, 2-aminoacetophenone, EtOH (52%); (**b**) conc. H_2_SO_4_/AcOH (75%); (**c**) *N*,*N*-dimethylformamide diethyl acetal, DMF; then NH_4_Cl, AcOH (41% over two steps).

In a closely related approach, the same authors synthesized eilatin (**90**) starting from quinolone-5,8-dione **98** bearing a trifluoroacetyl-protected amino group at the phenyl ring. Regioselective CeCl_3_-catalyzed oxidative amination of **98** and 2-aminoacetophenone furnished, obviously under spontaneous *N*-deprotection/cyclocondensation, the tetracyclic aminoquinone **99**. After an acid-catalyzed cyclization step, which afforded hexacyclus **100**, eilatin (**90**) was obtained in a one-pot annulation of ring G using *N*,*N*-dimethylformamide diethyl acetal and ammonium chloride (Scheme 16) [32].

**Scheme 17:**
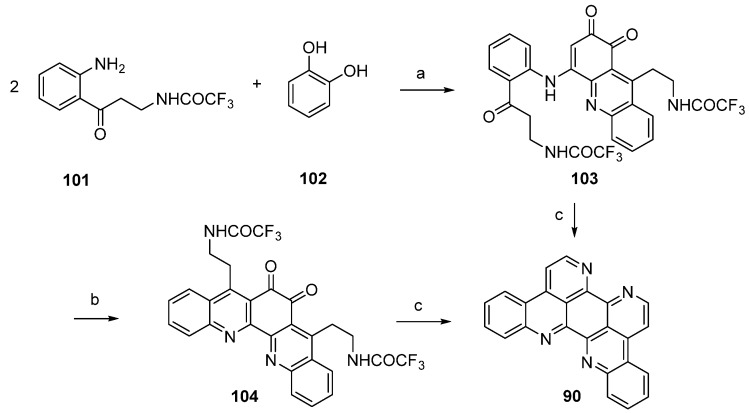
Biomimetic synthesis of eilatin (**90**): (**a**) NaIO_3_, EtOH (15%); (**b**) BF_3_·OEt_2_, CH_2_Cl_2_ (no yield given); (**c**) NH_3_, MeOH (no yield given) [34].

Kashman’s biomimetic approach towards eilatin (**90**) suggests that kynuramine and *o*-benzoquinone (or the corresponding catechol, **102**), both natural products, can be considered as potential biosynthetic precursors of this alkaloid. In the first step, the monoprotected trifluoroacetyl kynuramine **101** was reacted with catechol (**102**) under oxidative conditions to give compound **103** in very low yield. Treatment of **103** with ammonia in methanol and DMAP directly furnished alkaloid **90**. An alternative route towards eilatin (**90**) was accomplished by the treatment of **103** first with BF_3_ etherate. The so-obtained pentacyclus **104** was converted to eilatin (**90**) under alkaline conditions (NH_3_-MeOH) (Scheme 17) [34]. No yields were given for the final steps in both approaches.

**Scheme 18:**
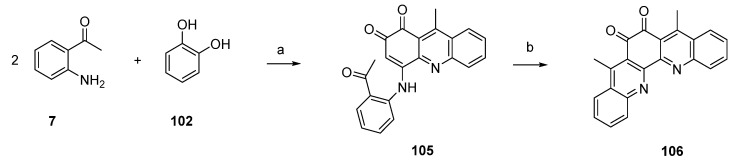
Synthesis of postulated eilatin precursor **106**: (**a**) NaIO_3_, EtOH (60%); (**b**) BF_3_·OEt_2_, CH_2_Cl_2_ (70%).

In this and an accompanying paper [35] the Kashman group described several unsuccessful approaches to eilatin (**90**). One of these attempts is illustrated in Scheme 18. In a conversion closely related to the one described in Scheme 17, compound **105** was prepared from 2-aminoacteophenone (**7**) and catechol (**102**) under oxidative conditions. Ring closure was accomplished by treating **105** with BF_3_-etherate and this furnished the symmetrical pentacyclic dibenzo-1,10-phenanthroline-5,6-dione **106** in high yield. A completion of this synthesis (annulation of rings F and G of eilatin) has not yet been published.

**Scheme 19:**
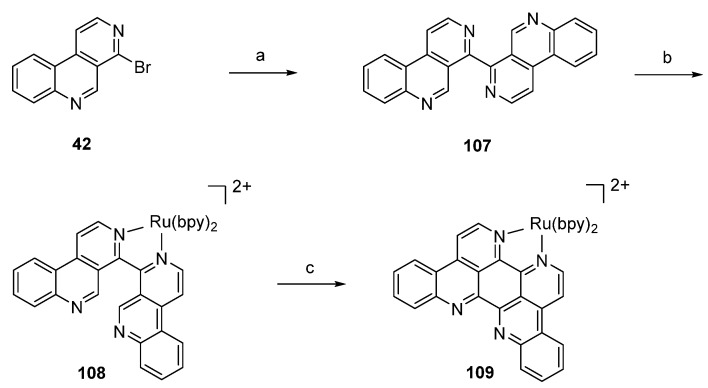
Synthesis of Ru^II^-eilatin complex **109**: (**a**) Pd(OAc)_2_, Bu_4_NBr, K_2_CO_3_, *i*PrOH, DMF (**b**) Ru(bpy)_2_Cl_2_·5H_2_O, ethylene glycol, water (62%); (**c**) Pd/C, ethylene glycol-acetone (97%).

The synthetic strategy employed by Glazer and Tor [36] for the preparation of a Ru^II^-eilatin complex started with “pre-eilatin” (**107**), a formal seco analogue of the alkaloid, which was obtained in 54% yield by Pd-catalyzed homo-coupling of 4-bromobenzo[*c*][2,7]naphthyridine (**42**). Treatment of “pre-eilatin” (**107**) with Ru(bpy)_2_Cl_2_·5H_2_O in ethylene glycol and water gave the dark-red Ru^II^ complex **108** ([Ru(bpy)_2_(pre-eilatin)]^2+^), which was converted into the deep-green [Ru(bpy)_2_(eilatin)]^2+^ complex **109** by exposure to palladium on carbon in ethylene glycol and acetone at elevated temperatures in almost quantitative yield. Separation of free eilatin from the complex **109** is not mentioned in the publication. It is noteworthy that treatment of the free “pre-eilatin” (**107**) ligand with Pd/C did not yield eilatin (**90**) (Scheme 19). This approach is quite short, but affords stoichiometric amounts of the expensive Ru complex for the key step.

**Scheme 20:**
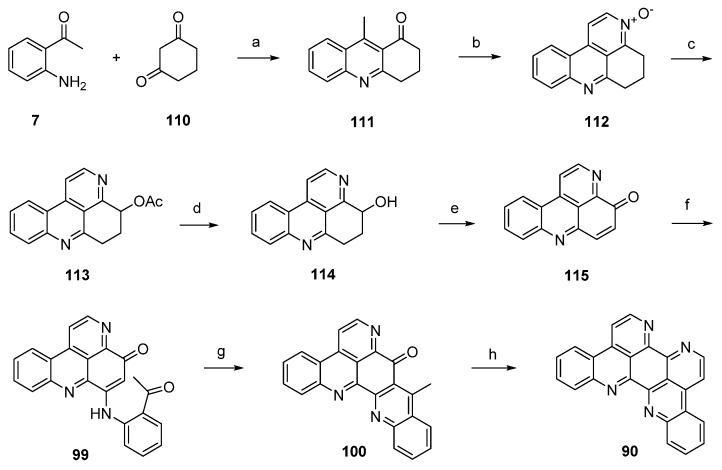
Synthesis of eilatin (**90**): (**a**) Reflux (54%); (**b**) *N*,*N*-dimethylformamide diethyl acetal, DMF; then NH_2_OH·HCl (41% over two steps); (**c**) acetic anhydride (66%); (**d**) 10% NaOH in H_2_O/MeOH (84%); (**e**) MnO_2_ (66%); (**f**) 2-aminoacetophenone, CeCl_3_·7H_2_O (74%); (**g**) 10% H_2_SO_4_ in AcOH (96%); (**h**) *N*,*N*-dimethylformamide diethyl acetal, DMF; then NH_4_Cl (65% over two steps).

A divergent synthesis leading to both eilatin (**90**) and its isomer isoeilatin (**119**) was published by Plodek and Bracher in 2015 [37]. This approach started with a Friedländer reaction of 2-aminoacetophenone (**7**) and 1,3-cyclohexanedione (**110**) to afford acridone **111**. Following one-pot annulation of a pyridine-*N*-oxide ring was accomplished by condensation of the acidic methyl group of **111** with *N*,*N*-dimethylformamide diethyl acetal under controlled conditions (55 °C) and subsequent ring closure with hydroxylamine hydrochloride to give *N*-oxide **112**. In a Boekelheide rearrangement, this *N*-oxide was converted to the acetoxy compound **113** by heating with acetic anhydride. Alkaline ester hydrolysis of **113** afforded alcohol **114** in 84% yield. Subsequent oxidation of this alcohol under mild conditions with MnO_2_ under concomitant ring dehydrogenation furnished pyridoacridone **115** in 66% yield. Further conversion to eilatin (**90**) was performed in analogy to Bracher’s total synthesis of ascididemin (**3**) [7]. Thus, regioselective oxidative amination of **115** with 2-aminoacetophenone under CeCl_3_ catalysis gave arylamino derivative **99**. Next, acid-catalyzed cyclization furnished hexacyclic compound **100** in almost quantitative yield. The final annulation of ring G was performed in a one-pot reaction by condensation of the acidic methyl group with *N*,*N*-dimethylformamide diethyl acetal, followed by heating with ammonium chloride/glacial acetic acid to give **90**. Hence, alkaloid **90** was synthesized in seven steps with an overall yield of 6.9% from only two building blocks (1,3-cyclohexanedione, 2-aminoacetophenone) (Scheme 20) [37].

**Scheme 21:**
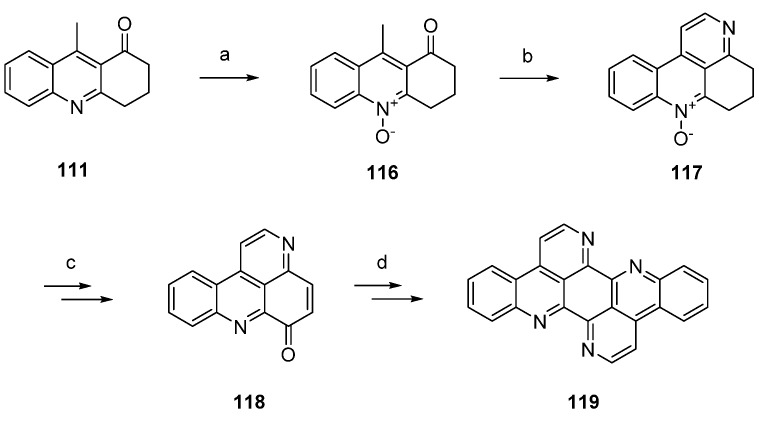
Synthesis of isoeilatin (**119**): (**a**) *m*CPBA, CH_2_Cl_2_ (88%); (**b**) *N*,*N*-dimethylformamide diethyl acetal, DMF; then NH_4_Cl (38% over two steps); (**c**) acetic anhydride (80%); then 10% NaOH in H_2_O/MeOH; then MnO_2_, toluene (50% over two steps); (**d**) 2-aminoacetophenone, CeCl_3_·7H_2_O; then 10% H_2_SO_4_ in AcOH; then *N*,*N*-dimethylformamide diethyl acetal, DMF; then NH_4_Cl (38% over four steps).

Isoeilatin (**119**), a synthetic isomer of **90**, was prepared from the same building blocks by using different reaction conditions. In this synthesis, acridone **111** was first treated with *meta*-chloroperoxybenzoic acid (*m*CPBA) to furnish *N*-oxide **116**. An annulation of a pyridine ring through condensation with *N*,*N*-dimethylformamide diethyl acetal and subsequent ring closure with ammonium chloride gave tetracyclic compound **117**, an isomer of the *N*-oxide **112** utilized in the synthesis of eilatin (**90**), in 38% yield. In analogy to the above-mentioned protocol, *N*-oxide **117** was subjected to Boekelheide rearrangement, ester hydrolysis and oxidation with MnO_2_ to furnish pyridoacridone **118**. The following steps (oxidative amination, acid-catalyzed cyclization, annulation of the seventh ring using *N*,*N*-dimethylformamide diethyl acetal and ammonium chloride) were performed in full analogy to the above-mentioned total synthesis of eilatin (**90**) [37]. Isoeilatin (**119**) was obtained in eight steps with 5.1% total yield (Scheme 21) [37].

**Scheme 22:**
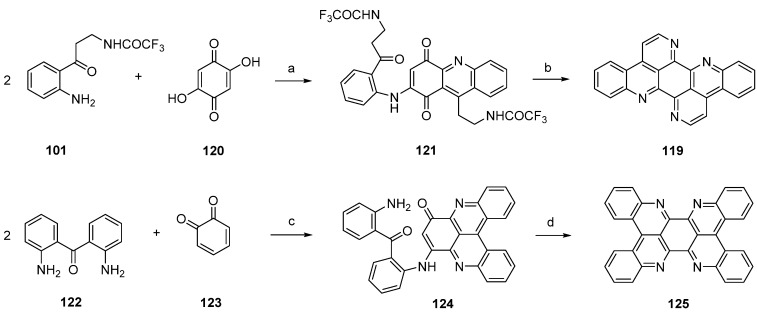
Synthesis of isoeilatin (**119**): (**a**) AcOH/Et_3_N (7%); (**b**) NH_3_ in methanol (43%). Synthesis of dibenzoeilatin (**125**); (**c**) CeCl_3_·7H_2_O, EtOH (36%); (**d**) AcOH/H_2_SO_4_/Et_3_N (no yield given).

Another approach towards isoeilatin (**119**) was reported by Kashman [38] in a paper published in 1994. Reaction of monoprotected trifluoroacetyl kynuramine **101** and 2,5-dihydroxy-1,4-cyclohexanedione (**120**) under acidic conditions (AcOH/Et_3_N) furnished isoeilatin precursor **121** in 7% yield. Following treatment of with ammonia in methanol provided isoeilatin (**119**) (Scheme 22).

The authors also described the synthesis of a dibenzo analogue of eilatin (**125**) in this publication [38]. For this purpose, 2,2′-diaminobenzophenone (**122**) was reacted with *o*-benzoquinone (**123**) in the presence of CeCl_3_ to give compound **124**. Subsequent treatment with acid (AcOH/H_2_SO_4_/Et_3_N) of **124** afforded the highly symmetrical “dibenzoeilatin” (**125**) (Scheme 22) [38].

## 5. Styelsamine- and Cystodytin-Type Pyridoacridines

**Figure 5:**
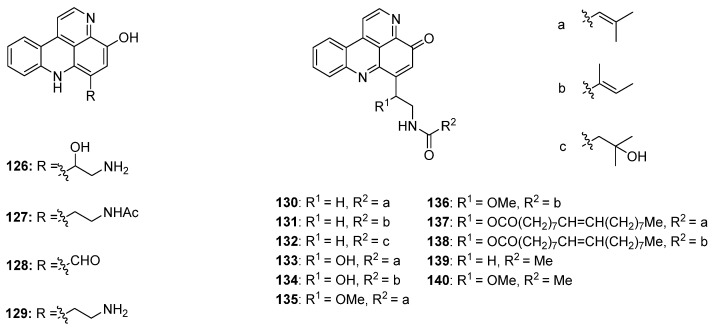
Structures of the styelsamines A–D (**126**–**129**) and cystodytins A–K (**130**–**140**).

Styelsamine- and cystodytin-type pyridoacridines are teracyclic alkaloids bearing a functionalized side-chain at C-6. In contrast to cystodytin-type alkaloids, which possess the pyrido[4,3,2-*mn*]acridin-4-one skeleton, styelsamine alkaloids are characterized by the hydroxylated pyrido[4,3,2-*mn*]acridine core. Until today, four stylesamine-type (stylesamines A–D; **126**–**129**) [39] and 11 cystodytin-type alkaloids (cystodytin A–K; **130**–**140**) [40,41,42,43] have been isolated and characterized (Figure 5).

**Scheme 23:**
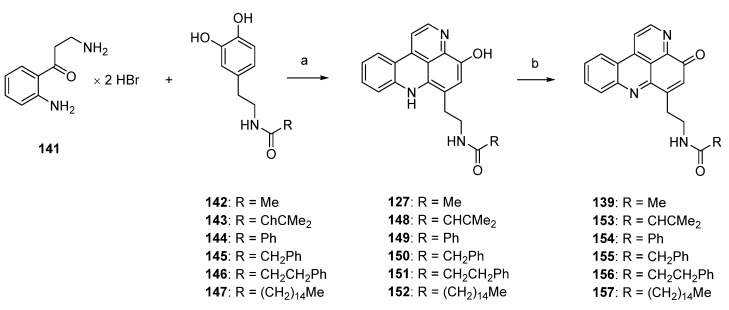
Synthesis of styelsamine (**127**, **148**–**152**) and cystodytine analogues (**139**, **153**–**157**): (**a**) CeCl_3_·7H_2_O, Ag_2_O, MeOH/AcOH (2:1), then 6 M HCl (6%–20%); (**b**) Ag_2_O (one equiv.), MeOH (13%–79%).

Based on Skyler and Heathcock’s biomimetic approach [44] to stylesamine B (**127**) and cystodytin J (**139**), Fong and Copp reported the synthesis of a series of side-chain substituted analogues of styelsamine and cystodytin alkaloids in 2013 [45]. The key step of this biomimetic strategy is a CeCl_3_-catalyzed oxidative coupling of kynuramine (**141**) and functionalized dopamine analogues **142**–**147** in the presence of silver oxide. While kynuramine (**141**) was prepared from a *N*-protected tryptamine *via* oxidative cleavage of the pyrrole ring, the dopamine analogues **142**–**147** were synthesized from 3,4-dimethoxyphenethylamine by *N*-acylation and subsequent *O*-demethylation (both syntheses are not shown in Scheme 23). *In situ* oxidation of the catechol moiety of the dopamines with silver oxide gives *ortho*-quinones, which undergo CeCl_3_-mediated nucleophilic addition of the aromatic amino group of kynuramine (**141**) (the more basic aliphatic side-chain amino group is prevented from this reaction by protonation) and re-oxidation to an aminoquinone. Subsequent treatment with 6 M hydrochloric acid resulted in cyclization to the acridines and, finally, closure of the fourth ring in an imine formation/tautomerization sequence related to a protocol that is known from Kashman’s biomimetic ascididemin synthesis [13]. The stylesamine-type pyridoacridines **127**, **148**–**152** were isolated in yields of 6% to 20%. Subsequent oxidation of **127**, **148**–**152** with one equivalent of silver oxide in bicarbonate-buffered methanol furnished cystodytin-type pyridoacridines **139**, **153**–**157** in 13%–79% yields (Scheme 23) [45].

**Scheme 24:**
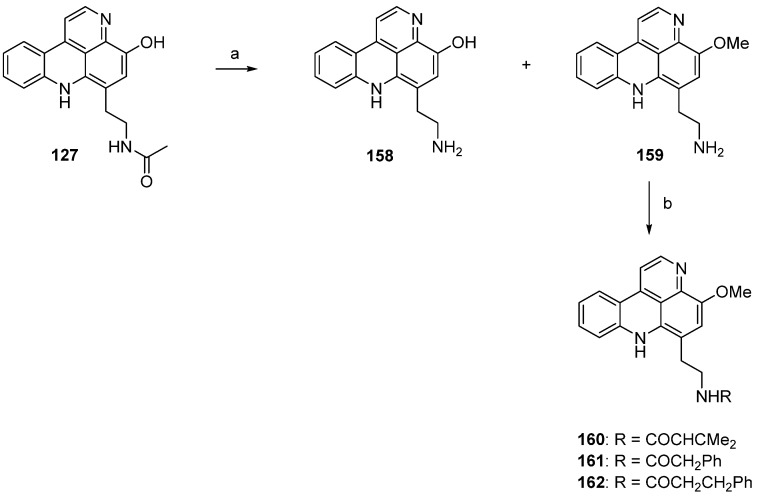
Synthesis of analogues of styelsamine alkaloids: (**a**) 4 M HCl/MeOH (1:1) (**158**, 60% and **159**, 45%); (**b**) for **160** and **161**: corresponding carboxylic acid, DMF, CH_2_Cl_2_, Et_3_N, PyBOP (**160**, 88% and **161**, 48%); for **162**: dihydrocinnamoyl chloride, THF, Et_3_N (43%).

In 2002, Skyler and Heathcock [3] reported that treatment of stylesamine B (**127**) with hydrochloric acid (4 M) in methanol afforded the alkylamino analogue of stylesamine D **158** in quantitative yield. Repeating this experiment, Copp isolated, besides the desired product **158**, the unexpected *O*-methyl analogue **159**. With this *O*-methyl analogue **159** in hand, the authors were able to prepare the *N*-acyl analogues **160**–**162**. While acrylamide **160** and 2-phenylacetamide **161** were synthesized by treatment of **159** with appropriate carboxylic acids in DMF and PyBOP, 3-phenylpropanamide **162** was prepared from **159** by reaction with dihydrocinnamoyl chloride in THF (Scheme 24) [45].

**Scheme 25:**
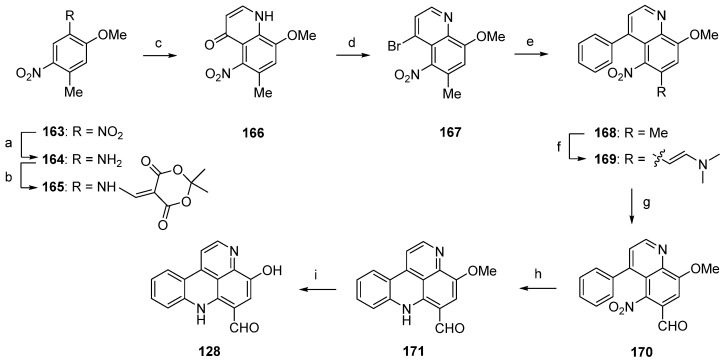
Synthesis of styelsamine C (**128**): (**a**) 10% Pd/C, cyclohexene/EtOH (60%); (**b**) Meldrum’s acid, trimethyl orthoformate (94%); (**c**) diphenyl ether, reflux (83%); (**d**) POBr_3_ (78%); (**e**) phenylboronic acid, EtOH/toluene, K_2_CO_3_, Pd(PPh_3_)_4_ (94%); (**f**) *N*,*N*-dimethylformamide dimethyl acetal, 170 °C (91%); (**g**) NaIO_4_, THF/H_2_O (90%); (**h**) P(OEt)_3_ (65%); (**i**) BBr_3_ in CH_2_Cl_2_ (86% over two steps).

In 2003, Nakahara and Kubo reported the first total synthesis of styelsamine C (**128**) [46,47]. This nine-step approach with an overall yield of 16% started with selective transfer hydrogenation of 5-methoxy-2,4-dinitrotoluene (**163**) using cyclohexene and 10% palladium on carbon catalyst to give 2-methoxy-4-methyl-5-nitroaniline (**164**). The following treatment with Meldrum’s acid furnished enaminone **165**, which was cyclized in refluxing diphenyl ether to afford 4-quinolone **166** (47% yield over three steps). Reaction of **166** with phosphoryl bromide gave 4-bromoquinoline **167** in 78% yield. The 4-phenylquinoline **168** was obtained in almost quantitative yield by the Suzuki cross-coupling reaction of **167** with phenylboronic acid under standard conditions. The acidic methyl group at C-6 of the nitroquinoline **168** was converted to an aldehyde in two steps. Treatment of **168** with *N*,*N*-dimethylformamide dimethyl acetal at 170 °C (in the full paper [47] the authors present different conditions, *N*,*N*-dimethylformamide dimethyl acetal at 145 °C, but the same yield) furnished enamine **169** in 91% yield. Subsequent oxidative cleavage with NaIO_4_ in THF/water afforded aldehyde **170** in 90% yield. The final cyclization step was achieved *via* an intramolecular nitrene insertion reaction by the heating of **170** with triethyl phosphite, yielding the tetracyclic compound **171** in 65% yield. Styelsamine C (**128**) was finally prepared by *O*-demethylation of **171** with boron tribromide in 86% yield (Scheme 25) [46,47].

## 6. Sebastianine A

**Figure 6:**
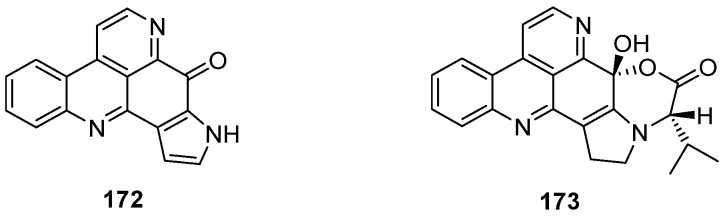
Structures of sebastianines A (**172**) and B (**173**).

Sebastianines A (**172**) and B (**173**) are members of a pyridoacridine subclass that possesses a pyrrole ring fused to ring A of the pyridoacridine ring system (Figure 6). In 2002, Torres *et al.* reported the isolation and structure elucidation of sebastianines A (**172**) and B (**173**) from the ascidian *Cystodytes dellechiaijei* [48].

**Scheme 26:**
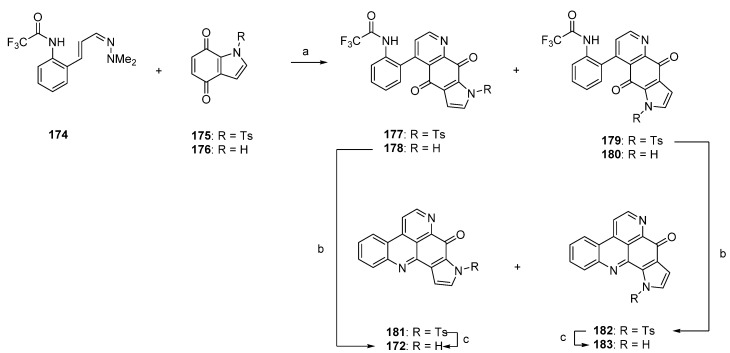
Synthesis of sebastianine A (**172**) and its regioisomer **183**: (**a**) Toluene, reflux, then MnO_2_ (no reaction conditions given) (R = Ts, 8%; R = H, 6%); (**b**) NaOH, CH_2_Cl_2_ (85%–92%); (**c**) NaOH, CH_2_Cl_2_ (95%–98%).

One year later, Delfourne published the first total synthesis of sebastianine A (**172**) [49]. This approach, which stands in close relationship to the above-mentioned synthesis of demethyldeoxyamphimedine (**56**) [12], again comprises a hetero-Diels-Alder cycloaddition as the key step. The required dienophiles, *N*-tosylindole-4,7-dione **175** as well as the corresponding unprotected indole-4,7-dione **176**, were obtained *via* 4,7-dimethoxyindole in multistep procedures (five and six steps). Cycloadditions with cinnanaldehyde-derived 1-azadieene **174** and subsequent re-aromatization with MnO_2_ gave mixtures of regioisomers **177**/**179** (5:95) or **178**/**180** (60:40) in very poor yields (6%–8%). Structures of the isomers were assigned only by analogy to the outcome of related cycloadditions performed by another group. Subsequent cyclization of **178** under alkaline conditions afforded sebastianine A (**172**) in 85% yield, and its regioisomer **183** was obtained in high yield by prolonged treatment of *N*-tosyl intermediate **179** with NaOH under cyclization and subsequent *N*-detosylation. (Scheme 26) [49]. The more complex alkaloid sebastianine B (**173**) has not been synthesized until today.

## 7. Arnoamine-Type Pyridoacridines

**Figure 7:**
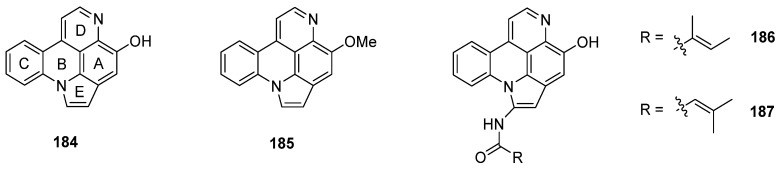
Structures of arnoamines A (**184**), B (**185**), C (**186**), and D (**187**).

Arnoamines A–D (**184**–**187**) are a unique type of pyridoacridine alkaloids, since they bear a pyrrole ring fused to rings A and B of the pyrido[4,3,2-*mn*]acridine skeleton. Isolated in 1998 (arnoamines A and B) and 2013 (arnoamines C and D) from two different ascidians (*Cystodytes* species), these pentacycles are up to today the only known representatives of this pyridoacridine subclass (Figure 7) [50,51].

The first total syntheses of arnoamines A (**184**) and B (**185**) were reported in 2000 [52] and have already been described in Delfourne’s review [6]. The key steps of this approach were the thermolysis of an arylaminomethylene in Meldrum’s acid derivative for construction of the quinoline scaffold (rings A and D), a Suzuki cross-coupling reaction of a functionalized 4-bromoquinoline (**188**) for the introduction of the phenyl ring (C), annulation of the pyrrole ring *via* Fischer indole synthesis, followed by cyclization under intramolecular *N*-arylation of the pyrrole ring (E) [52].

**Scheme 27:**
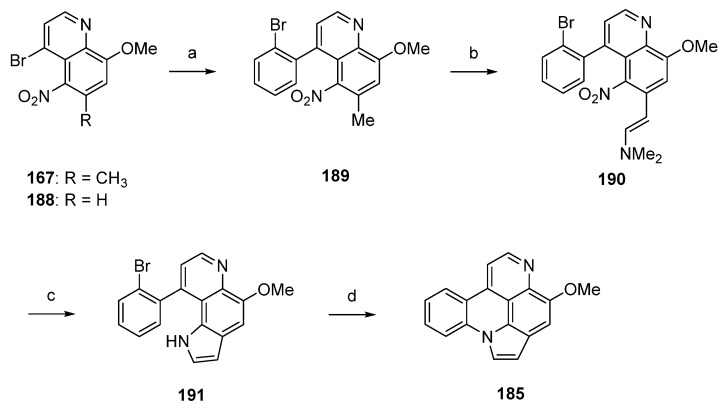
Synthesis of arnoamine B (**185**): (**a**) from **167**: 2-bromophenylboronic acid, H_2_O/toluene, K_2_CO_3_, Pd(PPh_3_)_4_ (85%); (**b**) *N*,*N*-dimethylformamide dimethyl acetal, DMF (83%); (**c**) Zn, AcOH/H_2_O (21%); (**d**) Pd(OAc)_2_, P(CMe_3_)_3_, K_2_CO_3_, xylene (81%).

A related approach to arnoamine B (**185**) was reported by Nakahara *et al.* in 2007 [53]. Starting from readily available 5-nitroquinoline **167** (see Scheme 25), alkaloid **185** was prepared in four steps with an overall yield of 12%. In the first step, **167** was subjected to a palladium(0)-catalyzed Suzuki cross-coupling reaction to give 4-(2-bromophenyl)quinoline **189** in 85% yield. Annulation of the pyrrole ring was accomplished in low yield (17% over two steps) utilizing the Leimgruber-Batcho protocol by condensation of the CH-acidic 6-methyl group of **189** with *N*,*N*-dimethylformamide dimethyl acetal to give enamine **190** and subsequent reductive cyclization with zinc powder in 80% aqueous acetic acid. Despite the low yield, this protocol is more convenient than Delfourne’s protocol starting from the des-methyl compound **188** and utilizing a Fischer synthesis for construction of the pyrrole ring. Finally, cyclization to arnoamine B (**185**) was accomplished in 81% yield under intramolecular *N*-arylation of the pyrrole moiety of **191** under palladium(II) acetate catalysis (Scheme 27) [53].

**Scheme 28:**
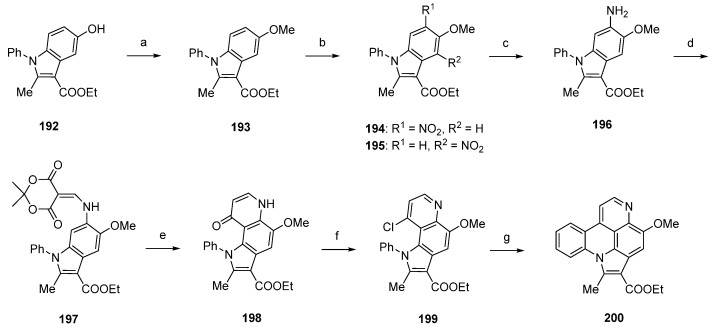
Synthesis of arnoamine B analogue **200**: (**a**) Me_2_SO_4_, 2 M NaOH, H_2_O, dioxane (95%); (**b**) HNO_3_, Ac_2_O (**194**, 63% and **195**, 30%); (**c**) from **194**: H_2_, Raney nickel, isopropanol (no yield given); (**d**) Meldrum’s acid, trimethyl orthoformate (95%); (**e**) diphenyl ether, reflux (80%); (**f**) POCl_3_ (96%); (**g**) Bu_3_SnH, AIBN, benzene (97%).

Using a different approach, starting from a *N*-phenylindole **192**, Radchenko *et al.* worked out a simple and effective route to the pyrido[4,3,2-*mn*]pyrrolo[3,2,1-*de*]acridine core of arnoamine-type pyridoacridines [54]. Synthesis of the arnoamine B analogue **200** started with *O*-methylation of **192** with dimethyl sulfate and sodium hydroxide to afford methyl ether **193** in almost quantitative yield. The following nitration with nitric acid (75%) in acetic anhydride furnished a mixture of nitroindoles **194** and **195** in 63% and 30% yield. Raney nickel-catalyzed hydrogenation of **194** gave primary amine **196**, which was converted to enamine **197** by treatment with Meldrum’s acid and trimethyl orthoformate in 95% yield. Subsequent thermal cyclization in refluxing diphenyl ether furnished pyrroloquinolone **198** in 80% yield. Arnoamine B analogue **200** was finally obtained by treating **198** with phosphoryl chloride (giving **199**) and subsequent radical intramolecular cyclization using tributyltin hydride and azobis(isobutyronitrile) in 93% yield over two steps (Scheme 28) [54].

**Scheme 29:**
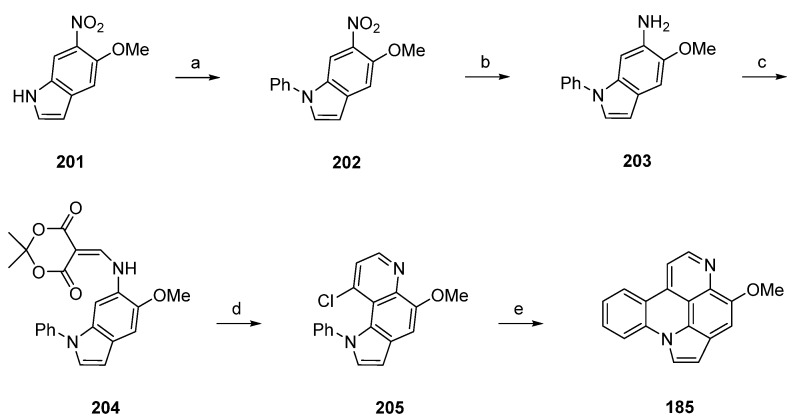
Synthesis of arnoamine B (**185**) by Kubo: (**a**) iodobenzene, CuI, (CH_2_NHMe)_2_, K_3_PO_4_, toluene (80%); (**b**) 10% Pd-C, methanol; (**c**) Meldrum’s acid, trimethyl orthoformate (66% over two steps); (**d**) Ph_2_O, reflux; then POCl_3_ (70% over two steps); (**e**) Bu_3_SnH (30 equiv.), AIBN (15 equiv.), toluene (90%).

In close relationship to this approach, Nakahara *et al.* achieved the synthesis of arnoamine B (**185**) in five steps with an overall yield of 33% [55]. This total synthesis starts with a CuI-catalyzed *N*-arylation reaction of 5-methoxy-6-nitroindole (**201**) with iodobenzene in toluene to give indole **202** in 80% yield. Following catalytic hydrogenation over 10% palladium on carbon in methanol yielded 6-aminoindole **203**. Annulation of the 4-pyridone ring was accomplished following the methods described above by treating **203** with Meldrum’s acid in trimethyl orthoformate (66% yield over two steps), followed by thermal cyclization of **204** upon heating in refluxing diphenyl ether. Subsequent treatment with phosphoryl chloride afforded chloro compound **205** in 70% yield over two steps. The final intramolecular cyclization step was performed using a large excess of tributyltin hydride and azobis(isobutyronitrile) in toluene to furnish the desired alkaloid **185** in 90% yield (Scheme 29) [55].

Arnoamines C (**186**) and D (**187**) have not been synthesized until today.

## 8. Subarine—A Formal Seco Analogue of Pyridoacridines

Subarine (**212**), a marine pyridyl benzo[*c*][2,7]naphthyridine alkaloid, is closely related to the pyridoacridine family, as it is formally a seco analogue of ascididemin-type alkaloids. This alkaloid was prepared following two different approaches by the Delfourne [56] and Bracher [57] groups.

**Scheme 30:**
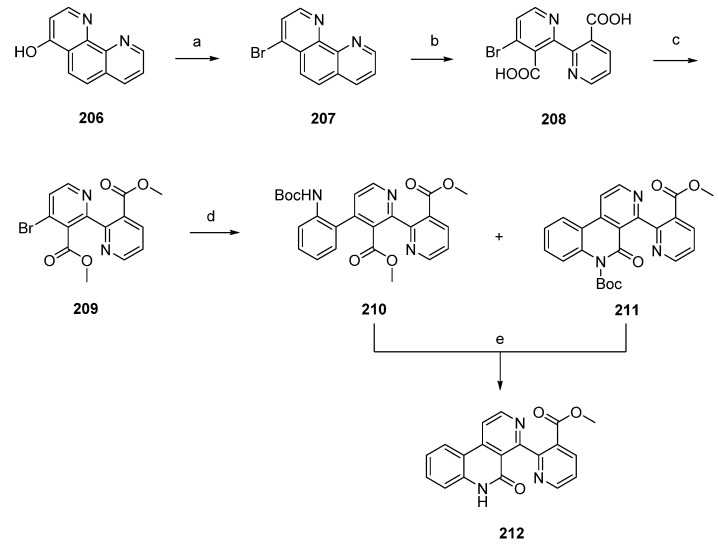
Synthesis of subarine (**212**) by Delfourne: (**a**) POBr_3_, PBr_3_ (79%); (**b**) KMnO_4_, KOH, H_2_O; (**c**) DCC, MeOH (88% over two steps); (**d**) 2-(NHBoc)C_6_H_4_SnMe_3_, Pd(PPh_3_)_4_, 1,4-dioxane (**210**, 63% ; **211**, 18%); (**e**) Et_3_N, CH_2_Cl_2_ (98% for both reactions).

Delfourne’s total synthesis of subarine (**212**) was accomplished in four steps with an overall yield of 70% starting from intermediate 1,10-phenanthrolin-4-ol (**206**), itself obtained in four steps from commercially available 8-aminoquinoline. Reaction of **206** with phosphoryl bromide and phosphorus tribromide gave the corresponding bromo derivative **207**. Subsequent oxidative cleavage of **207** with potassium permanganate under alkaline conditions afforded the binicotinic-type dicarboxylic acid **208**. The following conversion to diester **209** was accomplished by treatment of **208** with DCC and methanol. Palladium-catalyzed Stille cross-coupling reaction with *N*-(*tert*-butoxycarbonyl)-2-(trimethylstannyl)aniline afforded the expected phenyl-binicotinate **210**, together with the already cyclized lactam **211**. Subarine (**212**) was finally obtained by treating **211** with trifluoroacetic acid. In the case of **210**, synthesis was accomplished through cleavage of the Boc protecting group and a subsequent intramolecular cyclization reaction (Scheme 30) [56].

**Scheme 31:**
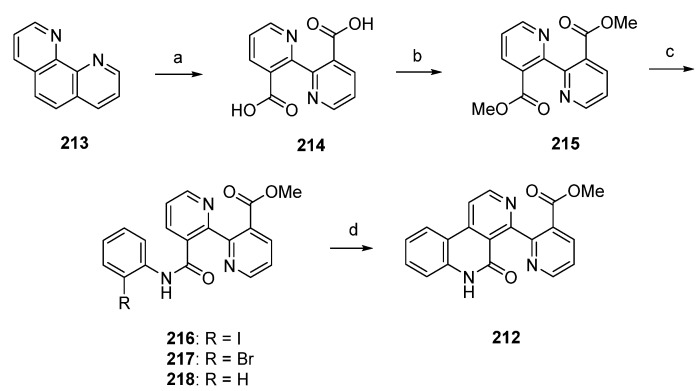
Synthesis of subarine (**212**) by Lotter and Bracher: (**a**) KMnO_4_, KOH, H_2_O (**b**) H_2_SO_4_, MeOH (73% over two steps); (**c**) 2-iodoaniline/2-bromoaniline, CH_2_Cl_2_, Me_3_Al, heptane (**216**, 40%; **217**, 35%); (**d**) Bu_3_SnH, AIBN, benzene (7% from **216**).

A significantly shorter synthetic route towards the alkaloid subarine (**212**) was reported by Lotter and Bracher in 2009 [57]. This four-step approach starts with the oxidation of 1,10-phenanthroline (**213**) using potassium permanganate. The so-obtained dicarboxylate **214** was esterificated by treatment with sulfuric acid in methanol to give the symmetric diester **215** in high yield. Amidation of **215** with 2-haloanilines under Weinreb conditions (trimethylaluminum, heptane) furnished the mono-2-haloanilides **216** and **217** in moderate yields, accompanied by the corresponding dianilides. The final cyclization step was accomplished by treating 2-iodoanilide **216** with tributyltin hydride and a catalytic amount of azobis(isobutyronitrile) in benzene to afford subarine (**212**) in very poor yield [57]. Significant amounts of deiodination product **218** were obtained in this reaction. Unfortunately, this approach gives only a very poor overall yield (Scheme 31).
